# “Folie à deux,” or shared psychosis: A case report

**DOI:** 10.1192/j.eurpsy.2024.1555

**Published:** 2024-08-27

**Authors:** E. Arroyo Sánchez, P. Setién Preciados, C. Diaz Mayoral

**Affiliations:** Hospital Universitario Príncipe de Asturias, Madrid, Spain

## Abstract

**Introduction:**

“Folie à deux,” or shared psychosis, is a fascinatingpsychiatric phenomenon characterized by the transmission of delusional beliefs and psychotic symptoms from one individual (the “inducer”) to another (the “recipient”) who share a close emotional bond. Despite its rarity, “Folie à deux” presents unique challenges and insights into the understanding of psychosis and the intricacies of interpersonal relationships.

**Objectives:**

The primary objective of this review is to analyze the recent clinical literature on “Folie à deux” to better comprehend its clinical presentation, diagnostic criteria, etiological factors, and therapeutic approaches. By synthesizing the latest research findings, we aim to enhance the awareness and understanding of this intriguing phenomenon among mental health professionals.

**Methods:**

A case report of a couple of a 34-year-old male and a 43-year-old female with a shared delirium. The male was brought to the emergency department by ambulance after being found in the street with behavioral disturbances and delusional symptoms. Individual interviews with both members of the couple revealed shared delirium. He was admitted to the psychiatric ward for the clinical picture consisting of a chronic delusional disorder of years of evolution and and new symptoms such as restlessness and behavioral disturbances.

**Results:**

The review reveals that “Folie à deux” remains a rare but clinically relevant phenomenon, with reported cases spanning diverse cultural and familial contexts. Diagnostic criteria, as outlined in the DSM-5, have been useful in guiding clinicians in identifying and managing cases. The literature emphasizes the importance of a thorough psychiatric evaluation to distinguish “Folie à deux” from other psychopathological conditions. Recent research has also shed light on potential neurobiological mechanisms and genetic factors contributing to shared psychosis. Therapeutically, early intervention and tailored treatment plans are crucial in achieving favorable outcomes. While antipsychotic medications remain a cornerstone of treatment, family therapy and psychoeducation have emerged as valuable adjunctive approaches to address the unique challenges posed by shared psychosis.

**Conclusions:**

In conclusion, “Folie à deux” continues to be a captivating and clinically relevant phenomenon in contemporary psychiatry. This bibliographical review underscores the importance of recognizing and diagnosing shared psychosis in clinical practice. Moreover, it highlights the need for further research to unravel the underlying mechanisms and genetic predispositions associated with this condition. Ultimately, a multidisciplinary approach, including pharmacological, psychotherapeutic, and family-based interventions, holds promise in improving the prognosis of individuals affected by “Folie à deux.”

**Disclosure of Interest:**

None Declared

